# A One Health Framework for Estimating the Economic Costs of Zoonotic Diseases on Society

**DOI:** 10.1007/s10393-012-0747-9

**Published:** 2012-03-07

**Authors:** Clare Narrod, Jakob Zinsstag, Marites Tiongco

**Affiliations:** 1 Joint Instirtute for Food Safety and Applied Nutrition, University of Maryland, College Park, MD USA; 2 Swiss Tropical and Public Health Institute, University of Basel, PO Box 4002, Basel, Switzerland; 3 International Food Policy Research Institute, 2033 K St, NW, Washington, DC 20006 USA

**Keywords:** one health, economic costs, zoonotic diseases

## Abstract

This article presents an integrated epidemiological and economic framework for assessing zoonoses using a “one health” concept. The framework allows for an understanding of the cross-sector economic impact of zoonoses using modified risk analysis and detailing a range of analytical tools. The goal of the framework is to link the analysis outputs of animal and human disease transmission models, economic impact models and evaluation of risk management options to gain improved understanding of factors affecting the adoption of risk management strategies so that investment planning includes the most promising interventions (or sets of interventions) in an integrated fashion. A more complete understanding of the costs of the disease and the costs and benefits of control measures would promote broader implementation of the most efficient and effective control measures, contributing to improved animal and human health, better livelihood outcomes for the poor and macroeconomic growth.

## Introduction

Zoonotic diseases are caused by many different pathogenic agents. In most cases, humans are accidental or “spill-over” hosts of a disease-ecological cycle maintained by animal hosts, including insects (Kayali et al. 2003; Schelling et al. 2003). Because of the circulation of zoonotic agents between animals, humans, and the environment, the cost of a disease affects human activity and health in addition to other economic sectors. According to the Institute of Medicine (2009), zoonotic pathogens caused more than 65% of emerging infectious disease events in the past six decades. The direct cost of zoonotic diseases over the last decade has been estimated to be more than $20 billion with over $200 billion indirect losses to affected economies as a whole (World Bank 2010). In the last 60 years, many industrialized countries have successfully controlled or eliminated zoonotic diseases through costly public investment facilitating coordinated interventions, including “test and slaughter,” feed bans, mass vaccination of domestic animals and wildlife, health education and milk pasteurization. These are highly effective methods of eliminating zoonotic diseases which require important operational, legal, and financial collaterals (Keusch et al. 2009). In most developing countries, surveillance of zoonotic diseases is not recognized as “one-health” collaboration between veterinary medicine and human medicine. In addition, many countries lack diagnostic capacity and health infrastructure. In livestock populations efforts have primarily focused on implementing prevention and eradication measures with much less emphasis on the effect of mitigation (transmission control) strategies, taking into consideration economic and development impacts at the macro (national economy, environment) or micro (health, livelihoods, food security of smallholder farmers) levels.

Many industrialized countries are able to control or reduce the risk of zoonotic diseases through public investment in preventative measures such as surveillance and compensation of farmers for culled stock in the event of an outbreak. In April 2001, the British government slaughtered and destroyed more than 2 million animals in England to stop the spread of foot-and-mouth disease (Sobrino and Domingo 2001). Such interventions are not feasible in many developing countries because of poor surveillance programs, limited institutional capacity, and, without donor assistance, lack of funds for livestock holder compensation (Zinsstag et al. 2007). This issue is illustrated by the limited effectiveness of the response following the HPAI outbreak in 2006–2008. Education programs to increase producer level bio-security measures were implemented in developing countries without careful consideration of how to alter behavior of small scale producers sustainably, despite high level ministerial support (Narrod et al. 2011). Successful investment in zoonoses control requires assessment of the cost of disease and the cost-effectiveness of proposed interventions, in addition to adaptation of the interventions to the local context. Given that 70% of the world’s rural poor depend on livestock and working animals for their livelihoods, animals cannot be left out of the solutions (LID 1999; FAO 2002).

Cost assessments of zoonoses require in-depth understanding of the ecology of disease. Detailed knowledge about transmission pathways helps identify sectors contributing to the cost of disease and is essential for determining effective interventions for interruption of the disease cycle. Zoonoses control is unique in that effective interventions may lie outside the health sector because transmission often does not occur between humans, but only from animals to human like in rabies or brucellosis (Zinsstag et al. 2005a, 2009b).

Economic impacts exist beyond the cost of control, including direct decreases in household income due to reduction in livestock/product sales, consumption impacts due to reduced food security, increased household vulnerability where livestock is used as a risk-coping mechanism and affects on household wealth which influence savings and gender equality (Birol et al. 2010). In addition there are impacts at the sector level, such as the feed and input sector or the broader economy which includes other analyzable input and output sectors (see You and Diao 2007; Diao et al. 2009). These associated costs may influence behavioral change at different levels (household, practitioners, policy) which is important to the decision-making process.

A “one health” approach demonstrates closer cooperation between human and animal health resulting in benefits that are not achieved through the two medicines working independently. “One health” evolved from “one medicine,” a term coined by veterinary epidemiologist Calvin Schwabe in the 1960s to demonstrate that there is no paradigm difference between human and veterinary medicine thus allowing for integrated work (Schwabe 1984). To date, there have been limited efforts to conduct integrated analyses considering both the social and ecological systems, although this approach is not conceptually new having been successfully applied in an “ecosystem approach to health” or “ecohealth” (Forget and Lebel 2001). We suggest that such an approach has enormous potential to improve public and animal health and provide cost savings in the public and private sectors. Sampling humans and animals simultaneously in an integrated study design decreases detection time for zoonotic disease (Schelling et al. 2003; Zinsstag et al. 2009a). Through integrated analysis, the full societal cost of disease can be estimated linking an animal–human transmission model to cross-sector economic analysis to show the full societal cost (Roth et al. 2003, Zinsstag et al. 2005a). The cost of livestock mass vaccination is often much higher than the public health benefit savings. Singularly from a public health perspective, such interventions are not cost-effective. An example is brucellosis control in Mongolia, where the intervention costs are less than a third of the overall cost of disease, when the private and agricultural sectors are included, with a societal benefit-cost ratio of 3.2 (Roth et al. 2003). Assessing the cost of zoonoses in multiple sectors facilitates identification of cost-sharing options such as a separable cost method. Although brucellosis control by livestock mass vaccination is not cost-effective from a public health sector perspective, it becomes highly cost-effective when costs are shared between the public health and agricultural sectors in proportion to their benefits (Roth et al. 2003). Integrated assessments are hence crucial for zoonotic disease control in resource poor countries (Zinsstag et al. 2007). The goal of the framework is to link the analysis outputs of animal and human disease transmission models, economic impact models, and evaluation of risk management options as a practical tool to gain improved understanding of factors affecting the adoption of risk management strategies so that investment planning includes the most promising interventions (or sets of interventions) in an integrated fashion.

## Proposed Framework

**Figure 1:**
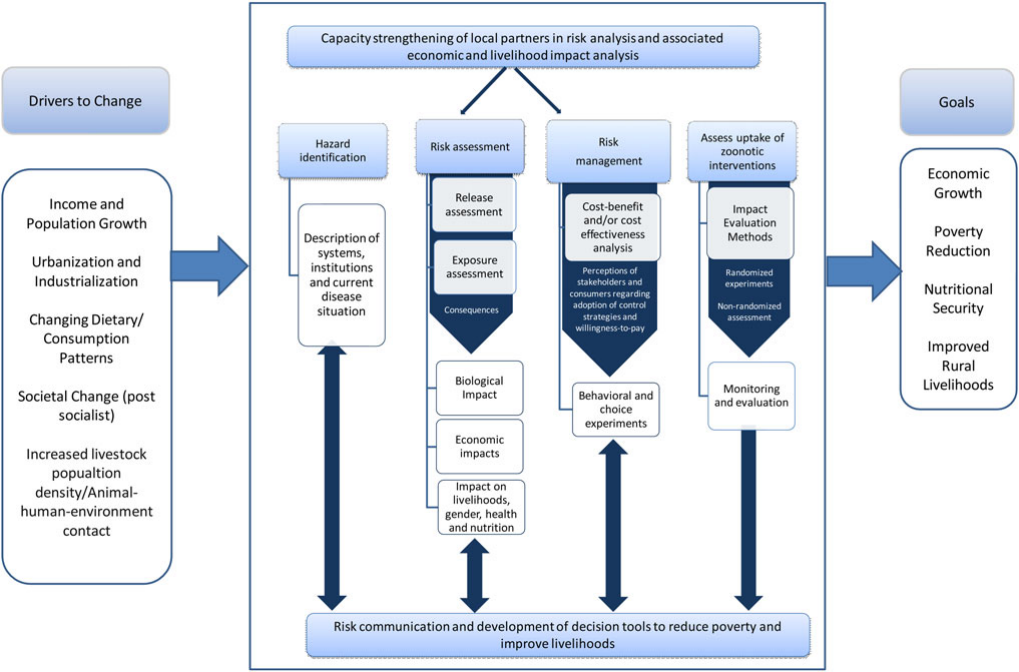
Modified risk analysis framework to enhance reduction of zoonotic disease burden.

The proposed “one health” framework is a modified risk analysis (Fig. 1) linking outputs associated with animal health transmission models, economic impact models, and risk analysis to inform the planning of investments through the most promising interventions (or set of interventions) and improve economic outcomes such as poverty alleviation, food security, and improved livelihoods. This framework allows identifying potentially useful types of analysis to inform decision makers prior to intervention implementation. This is valuable as decision makers evaluate different mitigation techniques to obtain a desired level of safety at a given cost. At best, mitigation is negotiated with all stakeholders, communities, authorities, and scientists in participatory transdisciplinary processes (Schelling 2008; Zinsstag 2007). Risk managers can choose strategies depending on the risk preferences for affected stakeholders and comparative advantages in implementing risk-reduction options. It is difficult to compare strategies which consider risk reductions and others evaluating costs and benefits. Despite good intentions, decisions can lead to losses in social welfare through unexpected outcomes and consequences. Decision makers would be aided by a framework which structures complex information and accounts for implications of the intricacy.

The proposed approach is similar to a traditional risk assessment, which includes a release assessment (where all potential pathways for disease introduction are identified), an exposure assessment (in which all potential pathways for exposure to the zoonotic disease in animals and humans are identified) and a consequence assessment. It is similar in that it also involves analysis to evaluate risk management efforts in terms of benefit costs and cost-effectiveness. A modification is that analyses enabling decision makers to consider stakeholders behavior modifiers, such as knowledge, attitude, and perception analysis and willingness to pay/adoption analysis, are also included. Additionally considered is an analysis enabling decision makers to understand factors affecting intervention uptake to assess successful strategies.

A stepwise approach is utilized:

I. Estimate the extent of the disease and potential spread;
II. Estimate the cost of zoonotic disease on livelihoods outcomes (income, health, and trade), including environmental impacts;
III. Assess the cost-effectiveness of risk management strategies currently employed for reduction of human and animal zoonotic disease exposure risk;
IV. Identify factors affecting adoption of zoonotic risk reduction strategies in poor households, the commercial sector and government bodies.

At all steps, participatory stakeholder consultations can take place which will ascertain perceived risk and mitigation priorities between all involved stakeholders.

Table 1 summarizes analytical methods for each step elaborating uses, strengths, and weakness, associated data requirements and possible users. Each proposed analytical approach has associated resource issues and it is not necessary to perform all simultaneously. Assembling the framework ensures that the analyses are integrated from the outset providing maximum benefit. Outputs of analytical efforts within the proposed framework will enable decision makers to evaluate the cost-effectiveness of various control measures and potential combinations for risk reduction from different perspectives. Calvin Schwabe’s “one medicine” concept has become more prominent in the last decade. The modified risk analysis approach described here correlates but has evolved towards “one health” conceptual thinking while emphasizing epidemiology and public health (Zinsstag et al. 2005b). Acceptance is reflected through adherence by professional organizations, governmental establishment of joint public and animal health working groups and inception of numerous research and surveillance programs (Zinsstag et al. 2009a, b). The proposed framework for estimating the societal cost of zoonoses is an open tool, translating the “one health” concept into practical methodology. It is consistent with the “One world one health” strategy, first defined in 2008, and currently adopted by the World Bank. The proposed framework is envisioned as a springboard for discussion, resulting in mutually adopted practical cooperation between human and animal health with a unique emphasis on developing countries but also global applicability (Zinsstag et al. 2009a).

**Table 1:** Joint human and animal research methods to assess zoonotic diseases

Methods/approaches	Uses	Strengths/weaknesses	Data needs	Users/agencies
Step I. Estimate the extent of disease and potential spread				
Joint human and animal disease frequency (Schelling et al. 2003; Bonfoh et al. 2011)	Identify the nature of the hazard and the source of infection. Policy makers to understand the full magnitude of the disease situation in a country/region	Enables stakeholders to understand magnitude of problem/cost of field studies and need for trained staff	Information on the nature and effects of hazards and exposure. Disease/pathogen occurrence, prevalence, and concentration	Animal and Public Health Ministries as well as private practitioners
Step II. Estimate the cost of zoonotic diseases on livelihoods outcomes				
Joint animal–human transmission dynamics (Zinsstag et al. 2005a, 2009b)	Understand nonlinear dynamics of transmission between animals and humans to simulate interventions	Ecological understanding of the animal–human transmission/needs good data, laborious and advanced understanding in mathematics	Time series of animal and human disease data (officially reported or actively collected)	Animal and Public Health Ministries
Macroeconomic impact (Roy 2008; Thurlow 2010)	Economic losses due to zoonotic diseases (shock vulnerability and resilience)	Macroeconomic understanding of effects of zoonotic diseases (social and societal level)/advanced econometric and economy-wide modeling expertise	Cross-sector time series data at the same level of aggregation	Ministries of Planning, Finance, Animal and Public Health
Livelihood analysis (Birol et al. 2010; Iannotti 2008; Roth et al. 2003)	Understand effects of zoonoses on poor households’ livelihood outcomes and nutrition	Effects of zoonotic diseases on livelihoods and household’s livelihoods/needs household surveys and trained staff	(Patient based) Household expenditure and consumption survey data.	Ministries of Finance, Animal and Public Health
Value chain and institutional analysis (Rich et al. 2011; Nguyen-Viet et al. 2009)	Understand relationship of risk and value chain to assess control mechanisms suitable for different actors along the value chain	Ecological understanding of critical risk control points along value and material flow chains/laborious in-depth understanding of value chain	Costs and returns at each stage of the value chain; flows and linkages of information and services	Ministries of Finance, Animal and Public Health Ministries, private sector
Step III. Assess the cost-effectiveness of risk management strategies currently used to reduce the risk of human and animal exposure to zoonotic diseases				
Cost benefit, cost-effectiveness analysis (Bennett 2003; Zinsstag et al. 2009b)	Understand cross-sector profitability and cost-effectiveness and risk–risk tradeoffs of various interventions	Cross-sector societal understanding of profitability of interventions/needs good data and advanced understanding of epidemiology, economics and mathematics	Social and private, direct and indirect economic costs and benefits of interventions; sales and net revenues	Ministries of Finance, Animal and Public Health, Practitioners, Private sector
Step IV. Identify the factors affecting adoption of zoonotic risk reduction strategies				
Knowledge, attitude, perception and practices (action), willingness to pay (Fielding et al. 2005; Di Giuseppe et al. 2008; Durr et al. 2008; Narrod et al. 2011)	Understanding actor’s knowledge, attitude, perceptions, practices towards controlling zoonotic diseases, and animal hosts and how it impacts practices	Cross-cultural understanding what actors perceive and motivates their actions and willingness to pay requires cultural science, expertise; not all control methods necessary in use in a developing country case; level of effectiveness of using risk reduction measures	Data on perception, knowledge, attitude, perception and practices (action), and willingness to pay	Animal and Public Health Ministries, private sector

Academics are users of all approaches and not specifically mentioned.

## Proposed steps

### Step 1: Estimating the Extent of the Disease and Potential Spread

#### Impact of Disease

Zoonoses cause human illness, permanent disability, and death. Animals may be asymptomatic carriers but can also be clinically ill or die. In livestock, illness may cause reduction in productivity, in numbers of live animals (reduced fertility) and reduced meat and milk production. The pooled impact of zoonoses on humans and animals to society can be estimated in terms of cost to different sectors.

##### Burden of Disease Estimate

Zoonotic diseases cause losses in goods produced (live animals, milk, meat, wool) and disability or loss of human life. The overall burden of disease to society involves a quantifiable monetary term and a quantifiable term reflecting loss of human life. Loss of human life can be quantified using standard life tables to sum the number of expected life years at the age of death. Non-fatal disease impairs human life during clinical illness and may result in temporary or permanent disability. WHO estimate the level of impairment of ill health and permanent disability related to complete physical and mental health and well-being (Disability weight = 0) and to death (Disability weight = 1). Disability weights of non-fatal diseases are classified depending on the level of impairment of human life to engage in occupation, procreation and recreation. This classification is controversial, raising ethical issues. Alternative ways of assessing the burden of disease address perceived quality of life, termed quality adjusted life years (QALYs). The proposed framework does not directly address this issue, instead focusing on the development of disability adjusted life years (DALYs) parameters, as currently in wide use, in order to increase the probability of effective interventions.

##### DALY Parameters

DALYs are used in the global comparative assessments of the burden of disease (Carabin et al. 2005) and enable costs of interventions to be related to a standardized health outcome across diseases internationally (Murray 1994; Murray and Acharya 1997). DALYs are a reflection of the time lived with a disability and the time lost because of premature death (Formula 1).

1 $$ {\text{DALYs }} = {\text{ years\;of\;life\;lost }} + {\text{ years\;of\;life\;with\;a\;disability}} $$

The duration of time lost due to premature death is calculated by using standard expected years of life lost with model life tables. The reduction in physical capacity due to illness is measured by using disability weights, mathematically expressed in Formula 2 (Murray and Acharya 1997)

2 $$ - \left[ {\frac{{DC{e}^{ - \beta a} }}{{\left( {\beta + r} \right)^{2} }}\left[ {{\text{e}}^{{ - \left( {\beta + r} \right)\left( L \right)}} \left( {1 + \left( {\beta + r} \right)\left( {L + a} \right)} \right) - \left( {1 + \left( {\beta + r} \right)a} \right)} \right]} \right] $$

where *a* is the age at onset of disease, *L* is the duration of disability or time lost due to premature mortality, *D* is the disability weight (or 1 for premature mortality), *r* is the discount rate, *C* is the age-weighting correction constant, and β is the parameter from the age-weighting function.

#### Methods for Estimating the Initial Prevalence of a Disease

Integrated methods, which investigate human and animal health simultaneously, are justified if the incremental knowledge generated is higher than two separate human and animal health studies, and if there are no concessions made with regard to the quality of methods used on either side. The interfaces between species can be straight forward or at different levels, e.g., by occupational or consumer exposure. In-depth assessments are then necessary to understand lifecycles and drivers of reservoir (maintenance host) populations. A variety of longitudinal and cross-sectional designs exist to monitor animal–human transmission using proxy indicators, for example, dog bites in the case of rabies (Cleaveland et al. 2002), questionnaires to determine exposure (Kayali et al. 2003) or comparative seroprevalence in human and potential animal reservoirs (Schelling et al. 2003; Zinsstag et al. 2009a). Studies at the animal–human interface should target high risk human populations within the context of exposure, such as encroaching habitat, live animal markets, or occupational risk groups (livestock workers, veterinarians) (Bonfoh et al. 2011).

### Step 2: Estimate the Cost of Zoonotic Diseases on Livelihoods Outcomes and National Economies, Including Environmental Impacts

#### Methods for Modeling Transmission

**Figure 2:**
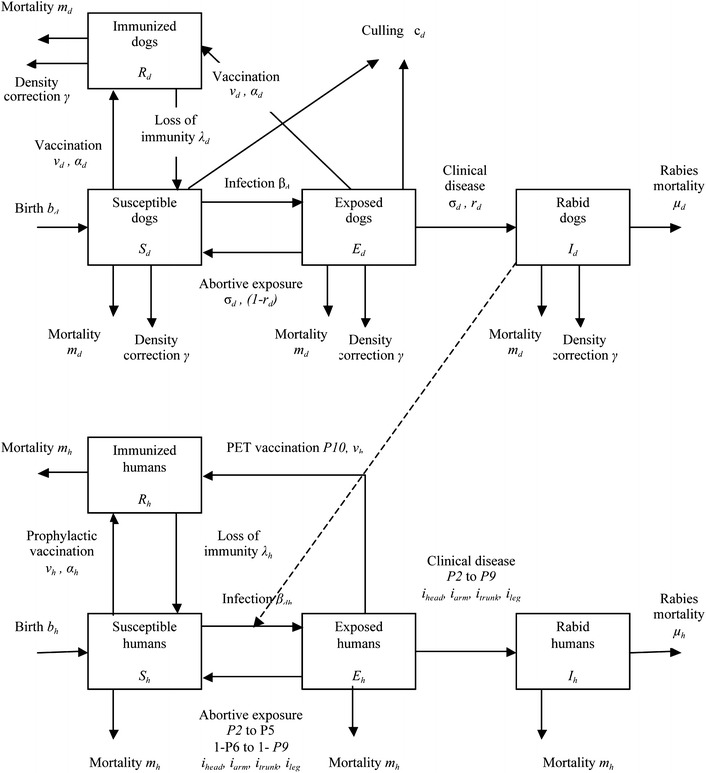
Flow chart of dog–human rabies transmission (Zinsstag et al. 2009b, with permission).

The cost and societal burden of zoonoses can be assessed in a static way from cross-sectional data. Additionally, benefit–cost analysis or cost-effectiveness of interventions can be done by comparing cost of disease before and after interventions, but these approaches do not consider the time-dependent dynamics of disease transmission with and without interventions. Zoonoses transmission can be endemically stable but usually undergoes epidemic cycles that are not captured by static approaches. Animal to human transmission is determined by the population dynamics. Animal–human transmission models are able to capture nonlinear dynamics in dissemination (Zinsstag et al. 2005a, 2006, 2009b), allowing human disease burden to be directly linked to the transmission in animals. A key feature of such models is that they can be used to simulate interventions, comparing outcomes with and without interventions (Fig. 2).

#### Assessing Effects on Livestock Productivity

Zoonoses affect the individual animal and herd productivity. Abortions reduce overall fertility of the herd, indirectly determining the number of live animals and production of meat and milk. To project effects of zoonoses on livestock production a livestock demographic model like the Livestock Development Planning System (LDPS; www.fao.org/agriculture/lead/tools/livestock0/fr, accessed September 2011), can be used (Roy 2008; Roth et al. 2003). It requires information about herd age and sex composition. This data can be obtained from national statistical offices or collected from large field surveys. Demographic models are driven by fertility and age-specific mortality. Fertility is expressed as number of newborn animals per female animal, in reproductive age per year. Age-specific mortality is the number of deaths per age group per year. Prior to simulating the effect of zoonoses on the demographic composition, baseline productivity should be simulated with known fertility and age-specific mortality data.

#### Methods for Modeling the Economic Cost of Disease

##### Macroeconomic Impact (Roy 2008)

The macroeconomic impact of zoonotic diseases can be modeled using a computable general equilibrium model or multi-market model. Model choice depends on livestock sector structure and the extent of structural linkages with other economy sectors and available data. Disease shocks like an occurrence of zoonosis can affect availability of livestock supply, for example through disease control measures such as eradication of infected animals reducing stock inventory. Declining production of livestock then affects household income through revenue losses for livestock keepers thereby affecting total national income, with decline in sales also influencing consumer prices.

Zoonotic disease outbreaks also impact the demand side through reduction in consumption expenditures on livestock products due to perceived food safety concerns or trade restrictions. This causes prices to drop, affecting producer livelihoods through lower returns causing diversion to non-livestock activities as compensation for falling returns from livestock. With non-livestock production increasing, prices for these non-livestock products fall, and thus benefiting other sectors in the economy. Similar to supply shocks, demand shocks also affect other sectors of the economy, including tourism. The net effect of the demand and supply shocks depends on income distribution and economy structure.

The models previously discussed use data from the national social accounting matrix, household budget survey, and household living standard survey and type of livestock commodity. If data are available at individual or farm level, a micro-simulation can determine the effect of disease shocks or risk mitigating/control measures on individuals’ income, wealth, and nutrition.

Macroeconomic models can be further integrated with available spatial disease spread models which reflect disease transmission. Spatial spread models are usually based on state and transition probabilities assessing the risk severity of disease outbreaks. Transition probabilities depend on transmission routes of infected livestock and trade flows (in country, cross-border) of the livestock products. To be useful, all data must be at the same aggregation level. In situations where actual data are not known, a series of simulations are projected using different levels of demand and supply shocks, e.g., varying dimensions of outbreak severity (minor: 15% to major: 30%), spread (local, nationwide) and duration (1–3 years). Economic losses can then be estimated across a wide range of scenarios, using no outbreak as a baseline.

Applications of this method using HPAI have been demonstrated (e.g., Thurlow 2010; Diao et al. 2009; Schmitz and Roy 2009). Economic losses due to avian influenza outbreaks and the effect on economic growth were estimated. Results suggested that demand shocks driven by consumer panic is the largest factor in reduction of poultry production, but the overall economic effect is likely to be minimal due to small size of the poultry sector and weak inter-sector linkages. The effect of an HPAI outbreak on rural poor income is not significant due to a diversified income portfolio with income from crops and other livestock contributing to shock resilience. The impact of HPAI on nutrition in Indonesian children was assessed by Iannotti et al. (2008). It was noted that reduced poultry product consumption resulting from a sustained HPAI shock without an animal origin food substitute would have significant detrimental impacts measured as growth stunting, height for age, and hemoglobin concentration for children (1–3 years old).

##### Microeconomic Impact

Both qualitative and quantitative analyses can be used to estimate the impact of zoonotic disease outbreak on income and wealth of households. Qualitative methods (focus group discussion, participatory rapid appraisal) are useful to understand the flow of livestock products along the value chain and identify bottlenecks, constraints or market failures and institutional risk management strategies (policies and regulations), as well as the social and political factors influencing livelihoods of impoverished households. The impact of economic losses on income generating activities, diversification patterns, and dynamic changes in income generating activities can also be investigated.

Quantitative analysis of costs, income, and consumption can be used to understand choices made by households and the effects on livelihood outcomes (increased income and food security). The impact of zoonotic diseases on household income and wealth can be estimated by measuring the changes due to supply and demand shocks and price changes with and without disease outbreaks. Data for this type of analysis may not be available without a household survey. In conducting a household survey, a counterfactual (without disease outbreak) scenario has to be identified against which the changes in livelihood outcomes (with disease outbreak) can be measured. This involves randomization of the sampling frame to maximize quantitative accuracy and eliminate selection bias. Where randomization is not possible, matching techniques, such as propensity score matching in which two groups of households with similar observable characteristics (household demographics, assets, income sources), can be used. The two household groups consist of a treatment group representing those with demand/supply shocks (with disease) and a control group representing the baseline (without disease). The differences between these groups in different scenarios of outcomes (income, productivity, wealth) reveal the impact of zoonotic disease outbreaks on income and wealth. Birol et al. (2010) used a similar approach to compare the impact of HPAI outbreak on livestock income and wealth by a scenario analysis.

### Step 3: Assess the Cost-Effectiveness of Control Strategies Currently Used to Reduce the Risk of Human and Animal Exposure to Zoonotic Diseases

#### Methods for Evaluation of Control Measures

Prevention and control strategies help minimize negative economic impacts of animal disease outbreaks, but there are costs associated with implementation. The costs and benefits of prevention and control measures must be assessed to inform policy makers for development of effective prevention and control policies.

##### Modeling the Direct Costs of a Disease

Effects of disease on livestock productivity (see above, assessing effects on livestock productivity) can be used to estimate direct cost of disease. The direct costs of the disease will be assessed using a partial budget model adapted from Bennett (2003). It is assumed that the direct costs of the zoonotic disease are additively related to loss in expected output, increase in expenditure on non-veterinary resources due to the disease and cost of inputs to prevent the disease.

#### Modeling Approach to Cost Benefit Analysis of the Intervention

The costs and benefits of the impacts of an intervention can be evaluated either in terms of public willingness to pay for them (benefits) or willingness to pay to avoid them (costs) or in terms of actual costs if control efforts have been implemented. Cost benefit analysis (CBA) is useful for governments to evaluate the desirability of a given intervention in markets. An intervention would be considered Pareto optimal if it improves the situation for some and does not worsen the situation of any. Pareto optimal solutions are difficult to achieve in practice. Potential Pareto solutions recognize that those who gain could compensate losers and still be better off and provide decision makers with a mathematical way to determine efficient interventions (Glauber and Narrod 2001). Acceptable intervention policies for governments are reflected when:

$$ E\left( {\text{Benefits}} \right) \ge E\left( {\text{Costs}} \right) $$

Though CBA traditionally focuses on efficiency by providing policy makers with an indication of the magnitude of net benefits associated with a particular policy, it is also possible to track the distribution of costs and benefits within different segments of the population. Ideally for zoonotic disease how costs and benefits are distributed by sector or geographic location would be determined. Therefore, the risk assessment should identify the higher risk pathways and sectors.

Because uncertainty and variability exists with all variables used in the CBA estimates it is important to conduct sensitivity and scenario analyses to illustrate how results change relative to the value of particular variables.

#### Cost-Effectiveness Analysis

Cost-effectiveness analysis aims to achieve the specified goal with the smallest loss in social welfare recognizing that the smallest loss might not be associated with the smallest financial cost. Towards analyzing control options associated with zoonotic diseases, the objective of the CEA analyses is to provide economic and disease risk and information on the impact of an intervention (or set of interventions). Certain strategies may have economies of scale which favor large producers.

**Figure 3:**
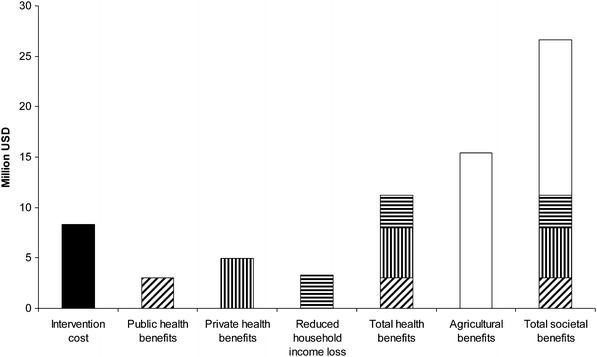
Costs and benefits of Brucellosis control in Mongolia (Roth et al. 2003). Intervention cost (*black*), public health benefits (*oblique lines*), private health benefits (*vertical lines*), reduced household income loss (*horizontal lines*), agricultural benefits (*white*).

Roth et al. (2003) estimated the societal economic benefit, cost-effectiveness, and distribution of benefit of improving human health through a brucellosis mass livestock vaccination campaign in Mongolia. A livestock-human brucellosis transmission model (Zinsstag et al. 2005a) was linked to a livestock productivity analysis to evaluate the impact of a planned 10-year livestock mass vaccination campaign to determine the cost-effectiveness, expressed as cost per DALY averted. The authors showed that if the costs of the intervention were shared proportional to the benefit to each sector, the public health sector would only contribute 11%, giving a cost-effectiveness of 19.1 USD per DALY averted (95% confidence interval 5.3–486.8). If private economic gain due to improved human health was included, the health sector would contribute 42% to intervention costs and cost-effectiveness would decrease to 71.4 USD per DALY averted. The conclusion was that if the costs of livestock vaccination were allocated to all sectors in proportion to the benefits, the intervention might be profitable and cost effective for the agricultural and health sectors (Roth et al. 2003). Figure 3 summarizes the costs and benefits of brucellosis control.

### Step 4: Identify the Factors Preventing the Adoption of Cost-Effective Strategies

#### Knowledge, Attitude, and Perception Analysis Surrounding Zoonotic Disease

Knowledge, attitude, and practice (KAP) analysis is increasingly used to evaluate the impact of education or intervention programs. The knowledge refers to the degree of understanding of the topic and associated issues, while attitude refers to respondent’s feelings towards them. Perception refers to the sense of awareness on the topic. Practices refer to past and current actions towards the topic. The KAP on zoonotic diseases has been investigated in general populations (Fielding et al. 2005; Di Giuseppe et al. 2008) and target groups (Abbate et al. 2006; Leggat et al. 2007). These studies used a Likert scale in the surveys, grouping questions into generalized groups where answers to each question were scored with points summed across. These KAP scores were then used to analyze the difference between different socioeconomic groups by univariate and/or multivariate analytical tools.

Recently Narrod et al. (2011) applied this approach to factors affecting knowledge about symptoms of avian influenza, attitudes on handling sick and dead birds, and perception of disease transmission in four countries in Africa. It was noted that production characteristics, relations with others and household characteristics influence individual’s knowledge, attitude, and perception and that in turn influences an individual’s behavior towards adopting specific biosecurity actions (practices).

##### Willingness to Pay/Adopt Analysis Surrounding Zoonotic Disease Control Analysis

Assessing public willingness to pay (WTP) is important in designing cost-effective measures to reduce disease risks and in estimating demand for these measures. Valid estimates of WTP for disease risk reduction are often used to inform the cost and benefits of technologies for prevention and control of zoonotic diseases. The economic values of the benefits of these technologies are not always known since most of these technologies are not yet market-available or adopted by consumers, so current prices may not reflect these benefits. To estimate a valuation of these non-market goods and to solicit consumers’ WTP for a product that is not yet on the market, economists have used contingent valuation (CV) methods originally developed in environmental and natural resource economics (Mitchell and Carson 1989). A hypothetical market is created for of the non-market good or service, contingent a non-market good or novel product, after which a group of subjects are invited to operate in that market and the results are recorded. The values generated through the use of the hypothetical market are treated as estimates of the value upon the particular hypothetical market (Mitchell and Carson 1989).

WTP can be estimated using open-ended questions, asking respondents to state the maximum amount they would be willing to pay, or dichotomous questions, asking the respondents if they would be willing to pay a specific amount or not. The open-ended format can be used when the consumer is well informed about the new product and its characteristics, but might not return realistic estimates if respondents do not have sufficient information to thoroughly consider the value attached to such goods if a market were to exist (Arrow et al. 1993). Dichotomous questions are easier for the respondent to assess and more realistic as they correspond to a usual market situation. In most markets, consumers are offered a product at a particular price and, perhaps after some bargaining, face a decision to purchase or not. Efficiency can be improved by offering the respondent a second bid, higher or lower depending on the first response, in an approach generally known as the double-bounded CV method (Hanemann et al. 1991). In this method, consumers will be given a hypothetical scenario involving the likelihood and severity of the outcomes, for example the number of people infected with rabies. Then consumers are presented with a price to see if they are willing to pay a certain amount for a definite safety level and, after responding yes or no, they are then presented with a second price bid, higher or lower than the first price. Finally, WTP can be modeled as a function of the severity and duration of illness, reduction in probability and respondent characteristics (Hammitt and Haninger 2007).

## Discussion and Conclusions

This article provides a comprehensive framework for assessing the societal cost of zoonotic diseases across all involved sectors. It is composed of novel joint methods to assess zoonotic disease frequence in animals and humans simultaneously, economic tools to estimate societal cost of disease and a mathematical framework simulating animal–human disease transmission, which can be used for comparative cost-effectiveness studies of interventions. For all parts case studies exist but only few studies exist that cover the whole range of the framework, e.g., a study on rabies in N’Djaména, Chad (Kayali et al. 2003; Durr et al. 2008; Zinsstag et al. 2009b). The importance of understanding the disease and host biology is highlighted because this is central to all control strategies. These assessments must be done in cooperation between epidemiologists, veterinarians, medical doctors, economists, anthropologists, and social scientists in the spirit of “one health”, benefiting from true closer cooperation across the human and animal health sectors (Zinsstag et al. 2005b, 2009a). The advantage of the framework is its potential for a comprehensive cross-sector societal assessment. However, it requires advanced capacity in epidemiology, economics, and mathematical modeling. As most of the steps require data collection, such an approach is costly and it may not always be feasible to undertake an exhaustive analysis simultaneously. It is suggested that research efforts be targeted at immediate needs, with additional analyses added over time to gain all information necessary for implementing effective control strategies which ensure the poverty alleviation and community participation. One of the critical issues are that most of the time household livelihood or patient-based private cost of disease studies are missing. They are, however, required, as private cost of disease is an important part of overall cost of disease, which is often higher than the public cost. Local perceptions, attitudes, and practices are often neglected because of the lack of capacity in cultural and gender studies. The framework can be used as modules, or in a reduced form using static instead of dynamic models. In this way approximations can be obtained with less resources and high level capacity. There remains, however, no doubt that governments in developing countries need to be informed as good as possible on the profitability and cost-effectiveness of interventions against zoonoses, in order to use scarce resources in the best way. Successful country specific zoonoses control is achievable over time within the framework. The framework’s approach has far reaching consequences because it includes all involved sectors. Cross-sector approaches may be needed not only when addressing health issues but also for environmental and societal problem solving.
